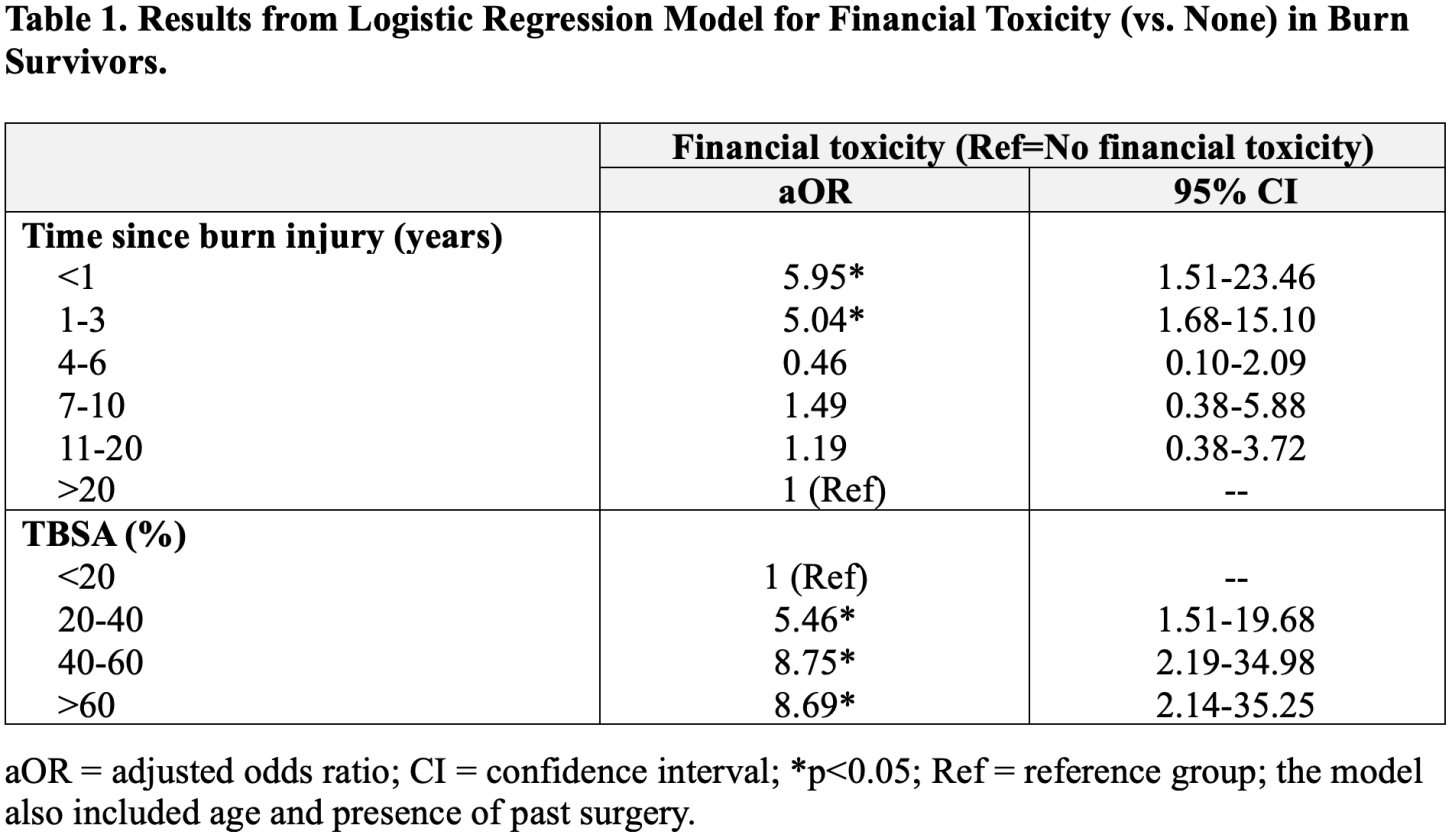# 501 A Nationwide Survey of Burn Survivors: Who Is at Greatest Risk for Financial Toxicity?

**DOI:** 10.1093/jbcr/iraf019.130

**Published:** 2025-04-01

**Authors:** Ana Reyes, Walter Ramsey, Christopher O’Neil, Michael Cobler-Lichter, Mary Ishii, Shevonne Satahoo, Joyce Kaufman, Louis Pizano, Tulay Koru-Sengul, Jose Szapocznik, Carl Schulman

**Affiliations:** University of Miami; University of Miami; University of Miami; University of Miami, Jackson Memorial Hospital; University of Miami Miller School of Medicine; University of Miami; University of Miami; University of Miami, Jackson Memorial Hospital; University of Miami Miller School of Medicine; University of Miami; University of Miami

## Abstract

**Introduction:**

The financial burden patients face due to healthcare costs is known as financial toxicity. Burn survivors are at high risk for financial toxicity given that their injuries can result in significant disability, lead to ongoing chronic medical issues, and often require treatment with multiple surgeries. Our study aim was to identify risk factors for financial toxicity in a nationwide sample of burn survivors. We hypothesized that financial toxicity would be more common in the first few years after burn injury.

**Methods:**

A survey was distributed through the Phoenix Society for Burn Survivors to adult burn survivors across the US (March-June 2023). The survey elicited demographics, burn history, treatment history, long-term difficulties faced, and unmet needs. We considered a survivor to have financial toxicity if they reported ‘financial assistance’ as one of their three greatest unmet needs or ‘impact of injury on employment/income opportunities’ as one of their three greatest long-term difficulties. Chi-square test for association and multiple binary logistic regression analyses were performed.

**Results:**

There were 178 respondents. The majority were female (60%), >54 years old (51%), White (75%), and had >20% total body surface area (TBSA) burns (75%). Twelve percent of survivors were < 1 year removed from injury, 17% 1-3 years, 11% 4-6 years, 7% 7-10 years, 14% 11-20 years, and 39% >20 years. In terms of prior procedures, 90% had surgery, 85% skin grafting, and 41% laser therapy. The rate of financial toxicity was 42%. On regression (Table 1) controlling for age, presence of past surgery, and TBSA, survivors who were < 1 year and 1-3 years from injury had significantly greater odds of financial toxicity compared to survivors who were >20 years from injury. Age and past surgery were not associated with financial toxicity.

**Conclusions:**

In this study, burn survivors ≤ 3 years from injury experienced the highest odds of financial toxicity. Having surgery did not incur a financial burden; however, this result may be biased given that most respondents were older White adults and lacked insurance data. Future studies should examine financial toxicity in more diverse populations.

**Applicability of Research to Practice:**

These findings may help guide prioritization of financial resources for burn injury survivors, with survivors ≤ 3 years from injury in most need of support.

**Funding for the Study:**

N/A